# Lycium Barbarum: A Traditional Chinese Herb and A Promising Anti-Aging Agent

**DOI:** 10.14336/AD.2017.0725

**Published:** 2017-12-01

**Authors:** Yanjie Gao, Yifo Wei, Yuqing Wang, Fang Gao, Zhigang Chen

**Affiliations:** Dongfang Hospital, Beijing University of Chinese Medicine, Beijing. China. 100078; Dongfang Hospital, Beijing University of Chinese Medicine, Beijing. China. 100078; Dongfang Hospital, Beijing University of Chinese Medicine, Beijing. China. 100078; Dongfang Hospital, Beijing University of Chinese Medicine, Beijing. China. 100078; Dongfang Hospital, Beijing University of Chinese Medicine, Beijing. China. 100078

**Keywords:** Lycium barbarum polysaccharides, Betaine, anti-aging, antioxidant, apoptosis, immunoregulation

## Abstract

Lycium barbarum has been used in China for more than 2,000 years as a traditional medicinal herb and food supplement. Lycium barbarum contains abundant Lycium barbarum polysaccharides (LBPs), betaine, phenolics, carotenoids (zeaxanthin and β-carotene), cerebroside, 2-O-β-d-glucopyranosyl-l-ascorbic acid (AA-2βG), β-sitosterol, flavonoids and vitamins (in particular, riboflavin, thiamine, and ascorbic acid). LBPs are the primary active components of Lycium barbarum. In this review, we discuss the pharmacological activities of LBPs and other major components. They have been reported to mediate significant anti-aging effects, through antioxidant, immunoregulative, anti-apoptotic activities and reducing DNA damage. Thus, the basic scientific evidence for anti-aging effects of LBPs is already available. However, additional studies are needed to understand mechanisms by which LBPs mediate anti-aging properties. Novel findings from such studies would likely pave the way for the clinical application of traditional chinese medicine Lycium barbarum in modern evidence-based medicine.

Lycium barbarum belongs to the family of Solanaceae. The berry is fusiform or oblong shaped with a length ranging from 6-20 mm and diameter 3-10 mm. The orange or dark red berry has a small stylar scar protruding from the top, and skin having shrunken appearance. The pulp is fleshy and soft with a bitter and sweet taste. The berry is eaten raw, consumed in juice form or added to tea or wine. The fruit is also processed to make tinctures, powders, and tablets. Moreover, it is used as food and medicinal plant in East Asia. Since the beginning of the century, the plant is commonly called as Goji in China. The other names include boxthorn, wolfberry, and Chinese wolfberry. The name of this plant is gugija in Korea and kuko in Japan. Lycium barbarum is mainly found in East Asia and grown particularly in South China, Korea, and Japan. The majority of commercially produced Lycium barbarum come from Ningxia Hui Autonomous Region in central North China and the Xinjiang Uyghur Autonomous Region in western China [1].

Lycium barbarum has been used for more than 2000 years in the traditional Chinese Medicine with early records traced back to the Tang Dynasty. Currently, it is part of the Pharmacopoeia of the People’s Republic of China. The recommended dosage of dried berries varies between 5 and 12 g. In Traditional Chinese Medicine, Lycium barbarum can treat various diseases, including blurry vision, abdominal pain, infertility, dry cough, fatigue, dizziness, and headache. Meanwhile, Lycium barbarum have long been used in Oriental medicine as a potent anti-aging agent. For instance, it is effective for counteracting premature graying of hair.

In modern medicine, some theories suggest that oxidative damage of biomolecules increases with age and is postulated to be a major causal factor of various aging-disorders. Immune dysregulation, irregulation of apoptosis and DNA damage are contributing factors to age-related pathologies and their associated morbidity and mortality. The concept of anti-aging by antioxidants such as Lycium barbarum has been supported by a line of evidence and has been investigated in different models [2, 3]. Meanwhile, Lycium barbarum has a variety of pharmacological functions, including immunoregulative, anti-apoptotic activities and reducing DNA damage, which can retard biological aging. Therefore, a large number of evidences suggest that Lycium barbarum is an effective anti-aging agent.

The leaves, fruits, and the root bark of Lycium barbarum contain abundant polysaccharides, carotenoids, flavonoids, alkaloids, amides, peptides, anthraquinones, coumarins, lignanoids, terpenoids, sterols, steroids, organic acids, anthocyanins, essential oils, and glycolipids. Lycium barbarum has a broad range of pharmacological activities, which is thought to be mainly due to its high concentration of Lycium barbarum polysaccharides (LBPs). In this review, we confer the anti-aging properties of Lycium barbarum constituents such as LBPs (comprising 5%-8% of the dried fruits), phenolics, stable vitamin C (Vc) analog AA-2βG, carotenoids (zeaxanthin and β-carotene), betaine, cerebroside, β-sitosterol, flavonoids, and vitamins (in particular, riboflavin, thiamine) [4]. Also, we critically deliberated the available scientific evidence for its anti-aging effects.

## 1. LBPs

### 1.1 The Anti-oxidant effects of LBPs

One of the major functional components of Lycium barbarum is the natural amino acid, Lycium barbarum polysaccharides, (LBPs). The LBPs contains several monosaccharides and 17 amino acids, including rhamnose (Rha), galactose (Gal), glucose (Glc), arabinose (Ara), mannose (Man), and xylose (Xyl) [5]. LBPs is a useful and an attractive candidate for counteracting oxidative stress and the various other detrimental factors associated with aging. The oxidative stress theory of aging is the most studied and accepted hypotheses for the molecular basis of aging. This theory was suggested that oxygen free radicals formed endogenously as byproducts from normal oxygen-utilizing metabolic processes can play an essential role in the aging process. Numerous studies have shown that oxidative damage increases with age in many organisms and that many different forms of Reactive oxygen species (ROS) may be the culprits of accumulated oxidative damage. This imbalance between prooxidants and antioxidants leads to the accumulation of oxidative damage to cellular macromolecules that increases during aging and contributes to the progressive decline in the function of cellular processes. Under this mechanistic framework, the regulation of oxidative stress may directly control the aging process.

#### 1.1.1 LBPs can scavenge free radicals and reduce oxidative stress reaction

Anti-oxidant mechanism is an important component of anti-aging. Oxidative stress refers to the oxidative and antioxidant imbalance, resulting in neutrophil inflammatory infiltration, protease secretion and a large number of intermediate oxidation products, including ROS and Reactive nitrogen species (RNS). ROS includes superoxide anion (O_2_-), hydroxyl radical (OH) and hydrogen peroxide (H_2_O_2_), etc. On the other hand, RNS includes nitric oxide (NO), nitrogen dioxide (NO_2_) and peroxynitrite (ONOO-).

A series of aerobic cells produce reactive ROS in the metabolic process. High concentration of ROS cause cellular oxidative stress, which may lead to cell senescence, apoptosis, and even necrosis. Studies also imply that some ROS regulate apoptosis and proliferation of tumor cells, and are associated with signal transduction between cells. Indeed, lower concentrations of free radicals can affect a series of signal transduction pathways that can lead to antioxidant activity in cells. It has been suggested that accumulation of oxidative stress will contribute to aging processes [6, 7]. Experiments showing the anti-aging effects of Lycium barbarum often use free-radicals as experimental toxic biomarkers.

One of the experimental models found that LBPs were efficient free radical scavengers. Studies in a model of aging found that LBPs were effective in scavenging free radicals such as O_2_- and OH [8]. LBPs inhibited malondialdehyde formation, scavenged O_2_-, and retarded superoxide formation in rat liver homogenate [9]. Another investigation showed antioxidant activity of LBPs in vivo. Ultraviolet spectrophotometry was adopted to determine the capability of LBPs to remove O_2_-, OH, 1, 1-diphenyl-2-picrylhydrazyl (DPPH) free radicals and Agreement of Basic Telecommunications Services (ABTS) free radicals. LBPs cleared O_2_-, OH and DPPH free radicals and its ROS clearance rate increased gradually with an increase in its concentration. However, when the concentration reached a certain value, the clearance rate leveled off [10]. Furthermore, LBPs could be used to compensate the decline in total antioxidant capacity, immune function and the activities of antioxidant enzymes. LBPs reduced the risks of lipid peroxidation accelerated by age-induced free radicals and antioxidant activities of LBPs were comparable to a normal antioxidant, vitamin C [11].

#### 1.1.2 LBPs enhance antioxidant enzyme activity

To protect against toxic effects of ROS and to modulate physiological effects of ROS, the cell has developed antioxidant defence systems. There are two types of antioxidant systems in the body. The first type of antioxidant enzyme system comprises enzymes such as superoxide dismutase (SOD), catalase (CAT), glutathione peroxidase (GSH-Px). The other type of antioxidant system is non-enzymatic, which includes ergothioneine, vitamin C, vitamin E, glutathione, melatonin, α- lipoic acid, carotenoids, trace elements copper, zinc, selenium (Se).

SOD, CAT and GSH-Px are synthesized in the body, which can eliminate detrimental molecules produced by the organism in the metabolic process. They are the primary free radical scavenging substance and the intuitive indicator for cell aging and cell death in vivo. SOD decomposes superoxide into hydrogen peroxide. CAT reduces hydrogen peroxide to water. GSH-Px reduces all organic lipid peroxides. SOD, CAT and GSH-Px comprise a complete oxidation chain. They can fight cell damage caused by oxygen free radicals as well as timely repair damaged cells. After body aging, the contents of SOD, CAT and GSH-Px decrease. Free radical scavenger decreases, and free radical increases and accumulates, causing unsaturated fatty acid oxidation generating a large number of lipid peroxides. Lipid peroxidation end product is Malondialdehyde (MDA), which has cytotoxicity and can increase the damage of membrane. But if supplement exogenous antioxidants, we can increase the content of SOD, CAT, GSH-PX in vivo and effectively inhibit free radical oxidative damage, and slow down aging.

LBPs increased antioxidase activities and reduced MDA level in diabetic rats [12]. Treatment with LBPs in a D-galactose induced mouse aging model resulted in enhanced lymphocyte proliferation and interleukin (IL)-2 activity, learning and memory ability and SOD activity of erythrocytes [13]. LBPs increased SOD, CAT and GSH-Px levels and reduced MDA concentration in the blood. It also improved skin SOD activity, reduced skin MDA content, and increased Hyp content [14, 15]. LBPs administration also significantly increased antioxidant enzyme activities and decreased creatine kinase activities. Antioxidant activity of LBPs has also been seen in humans. Thirty days intake of LBPs increased antioxidant efficacy in humans [16]. In summary, LBPs can increase the contents of antioxidant enzymes and decrease the content of MDA in the model aging group, indicating that it can protect tissues from the attack of oxidants and free radicals, thereby exerting its anti-aging effect.

#### 1.1.3 LBPs activate the Nrf2/ARE and Nrf2/HO-1 antioxidant pathways

Antioxidant/electrophile response element (ARE/EpRE)-regulated phase II detoxifying enzymes and antioxidants is one of the major antioxidant pathways involved in counteracting increased oxidative stress and maintaining the redox status in many tissues. Heme oxygenase-1 (HO-1), is one of the ARE-regulated phase II detoxifying enzymes and antioxidants, which are regulated by the redox-sensitive transcription factor nuclear factor erythroid 2-related factor (Nrf2).

Nuclear factor E2-related factor 2 (Nrf2) is the important role of the endogenous antioxidant system in vivo, which regulates the expression of detoxifying and antioxidant genes, such as heme oxygenase1 (HO-1), SOD, and CAT. In response to oxidative stress or pharmacological activation, Nrf2 is translocated into the nucleus and induces the expression of antioxidant enzymes by binding antioxidant response element (ARE). LBPs, a new PI3K/AKT/Nrf2 axis activator, prevented the development not only of oxidative stress but also of insulin resistance, as well as of glucose metabolic abnormalities. Activated Nrf2 of LBP represents a potential novel approach in the treatment and prevention of insulin resistance induced by Long-term high-fat diet [17].

Ultraviolet B (UVB) irradiation plays a key role in skin damage, which induces oxidative and inflammatory damages, thereby causing photoaging or photo-carcinogenesis. LBP was found to induce Nrf2 nuclear translocation and increase the expression of Nrf2-dependent ARE target genes. The antioxidant LBP could partially protect against UVB irradiation-induced photo-damage through activation of Nrf2/ARE pathway, thereby scavenging ROS and reducing DNA damage, and subsequently suppressing UVB-induced p38 MAP pathway. Thus, LBP can be potentially used for skincare against oxidative damage from environmental insults [18].

The Nrf2/HO-1 antioxidant pathway was adaptively activated to counteract the I/R-induced damage in the retina. LBP pretreatment did not only reduce the generation of ROS, but it also enhanced the activation of the Nrf2/HO-1 antioxidant pathway in I/R retinas. The Nrf2/HO-1 antioxidant pathway contributes to the protective effects of LBP in the rodent retina after I/R-induced damage. Pretreatment with LBP may also strongly potentiate the cell’s adaptive antioxidant ability [19].

### 1.2 LBPs markedly contribute to the immune modulation

The immune system is usually described at the organ, cellular and molecular levels regarding its components and function. It is classified as innate and adaptive immune systems. The immune system declines with age, which is termed “immunosenescence”, with changes likely in both innate and adaptive immunity. This senescence may decrease vaccine efficacy and lead to a higher incidence of infections, neoplasia, and autoimmune diseases. It has become increasingly evident that immune dysregulation is a contributing factor to age-related pathologies and their associated morbidity and mortality. Several studies have shown that the polysaccharides could upregulate both innate and adaptive immune responses. Thus, Lycium barbarum polysaccharide can markedly contribute to the immune modulation [20-23].

#### 1.2.1 The regulating effect of LBPs in innate immune

Innate immune response is an important part of body defense function which gradually formed in the long-term evolution process. This response comprises barrier function of normal tissue (skin, mucous membranes, etc.), bactericidal effect of body fluids, phagocytosis of macrophages and neutrophils, killing effect of the natural killer cells (NK) and natural immunity of cytokines.

##### LBPs can activate macrophages

Macrophages are a type of white blood cells that engulf and digest cellular debris, foreign substances, microbes, cancer cells, and anything else. Macrophages play pivotal roles in modulating immune function following infection, tissue injury and in tumor cytotoxity. As professional antigen presenting cells, macrophages help shape the innate and adaptive immune responses through the release of a variety of pro-inflammatory mediators at early time points following injury or infection. These cells also serve to dampen the inflammatory response through phagocytosis of apoptotic cells and secretion of soluble factors like IL-10. Age-related alterations in macrophage number and function have been correlated with enhanced susceptibility to infection, differential responses to tissue injury and enhanced tumor progression in rodent models [23].

LBPs can activate macrophages. LBPs markedly upregulated the expressions of CD40, CD80, CD86 and MHC class II molecules on peritoneal macrophages. LBP and LBPF1-5 activated transcription factors NF-kappaB and AP-1 by RAW264.7 macrophage cells, induced TNF-alpha, IL-1beta, IL-12p40 mRNA expression, and enhanced TNF-alpha production in a dose-dependent manner. Furthermore, LBPs significantly enhanced macrophage endocytic and phagocytic capacities in vivo. These results indicate that LBPs enhance innate immunity by activating macrophages. The mechanism may be through activation of transcription factors NF-kappaB and AP-1 to induce TNF-alpha production and upregulation of MHC class II costimulatory molecules [24]. Macrophages are principal immunostimulatory target cells of Lycium barbarum L. polysaccharide LBPF4-OL. LBPF4-OL prompts CD86 and MHC-II molecules expression on macrophages and greatly strengthen macrophage releasing of TNF-α and IL-1β [25]. Additionally, LBPL can enhance phagocytosis by the peritoneal macrophages and result in an enhanced NO production in mouse peritoneal macrophages [26].

##### LBPs promote the cytotoxicity of NK cells

NK cells are a type of cytotoxic lymphocytes critical for the non-specific immune response, which comprises killing of target cells without antigen sensitization, and without limit by the major histocompatibility complex (MHC). NK cells provide vital innate defenses against infections and malignancies using MHC independent cytotoxicity. Once activated, NK cells exhibit direct cytolytic activity on infected cells, also release pro-inflammatory cytokines and chemokines that contribute to the adaptive Th1 immune response. Age-related changes in both NK cell number and function in rodents have been described in the literature. Increased percentages of NK cells occur with advanced age, yet these cells exhibit decreased cytotoxicity on a per-cell basis. Cytokines and chemokines produced by NK cells in rodents decreases with advanced age [23].

LBPs can induce NK cells activation. LBPs markedly promoted the cytotoxicity of NK cells by enhancing interferon-γ (IFN-γ) and perforin secretion and increasing the expression of the activating receptor natural cytotoxicity receptor 30 (NKp30) under normal conditions. Meanwhile, polysaccharides can enhance NK cell function under simulated microgravity conditions by restoring the expression of the activating receptor NKG2D and reducing the early apoptosis and late apoptosis/necrosis. LBPs may be used as immune regulators to promote the health of the public and astronauts during space missions [27].

##### LBPs regulate function of dendritic cells

Dendritic cells (DCs) are antigen-presenting cells (also known as accessory cells) of the mammalian immune system. They play pivotal roles in the initiation of the primary immune response and act as messengers between the innate and the adaptive immune systems. Immature DCs are responsible for peripheral surveillance and phagocytosis of pathogens, and following antigenic uptake, these cells migrate to regional lymph nodes, stimulating T cell proliferation. Their role in modulating T cell effector function in the context of aging is an expanding field of interest. The dysregulation of one or more of the functions of DCs with advanced age can contribute to poor responses to infections and immunization, anti-tumor immunity and autoimmune pathology in the elderly [23].

LBPs are capable of promoting both the phenotypic and functional maturation of murine BMDC in vitro [28]. LBPs upregulated DC expression of I-A/I-E and CD11c, enhanced DC allostimulatory activity and induced IL-12p40 production. Maturation of DCs by LBPs able to directly activate the nuclear transcription factor NF-κB p65. The results revealed that LBP stimulation induces the phenotypic and functional maturation of DCs via TLR2- and/or TLR4-mediated NF-κB signaling pathways [29].

LBPs up-regulated the expression of CD40, CD80, CD86, and MHC class II molecules in DCs, down-regulated the uptake of Ag by DCs, enhanced DC allostimulatory activity, and induced IL-12p40 and p70 production [30]. LBPs also increased Th (helper T cell)1 response, and LBP-treated DCs enhanced Th1 and Th2 responses in vitro and in vivo. LBP in various clinical conditions to improve host immunity and suggested LBP as a potent adjuvant for the design of vaccines based on DCs [30].

##### LBPs could enhance cytokines secretion

Cytokines are important in cell signaling. It can be said that cytokines are involved in autocrine signaling, paracrine signaling and endocrine signaling as immunomodulating agents. Cytokines include chemokines, interferons, interleukins, lymphokines, and tumor necrosis factors. They act through receptors, and are particularly important in the immune system; cytokines modulate the balance between humoral and cell-based immune responses, and they regulate the maturation, growth, and responsiveness of particular cell populations. LBPL could significantly promote splenocyte proliferation synergistically with PHA or LPS, increase the ratio of CD4 (+) to CD8 (+) T cells and promote the cytokine secretion of macrophages; enhance protective effect of the maternally derived porcine circovirus type 2-specific (PCV2-specific) Immunoglobulin G (IgG) antibody responses, promote Th1 cytokines (IFN-γ and TNF-a) and Th2 cytokine (IL-4) secretion [31]. LBP able to activate chemokine receptor5 (CXCR5) + programmed death 1 (PD-1) + Follicular helper T cell (Tfh) cells and induce IL-21 secretion [32]. Lycium barbarum may also be useful substance for regulating the behavior, body weight and TNF-alpha [33].

#### 1.2.2 The role of LBPs in adaptive immune

Adaptive immunity is a part of body defense function when the body is in contact with the foreign antigen. Immune cells can select, recognize and clear these foreign antigens. Adaptive immunity includes cellular immunity and humoral immunity. LBPs can activate T cells. LBPs can be separated into five homogeneous fractions, designated LBPF1, LBPF2, LBPF3, LBPF4, and LBPF5. LBP, LBPF4, and LBPF5 significantly stimulated mouse splenocyte proliferation. The proliferation proved to be of T cells, but not B lymphocytes (B cells). LBP (i. p. or p. o.) administration significantly induced T cell proliferation. Activation of T cells by LBPs may contribute to one of its immuno-enhancement functions [34]. LBPs as an adjuvant could increase the generation of recombinant adenovirus vaccine-1 (rAd5VP1)-induced Tfh cells. LBPs might enhance T cell-dependent Ab responses by acting as an adjuvant for the generation of Tfh cells [32].

### 1. 3 LBPs inhibit apoptosis

Apoptosis is a cell autonomous orderly death for maintaining a stable internal environment, which is genetically controlled. Apoptosis is an active process involving gene activation, gene expression and gene regulation, and so on. Irregulation of apoptosis has been increasingly implicated in aging and aging-related diseases.

Apoptosis is a process controlled by multiple genes. These genes are B-cell lymphoma-2 family (Bcl-2 family), caspase family of oncogenes, P53 tumor suppressor gene and others. Abnormalities in the apoptotic process may be linked directly or indirectly with the occurrence of many diseases, such as cancer, autoimmune diseases. Radiation, drugs and many other factors can also induce apoptosis. The inhibition of apoptosis is closely related to the anti-aging, anti-oxidative stress approaches. So far, a variety of molecules have been found to have inhibitory effects on apoptosis including apoptosis P53, cytokine response modifier A (CrmA), FLICE inhibitory proteins (FLIPs) and Bcl-2 family of inhibitory molecules. Bcl-2 has the physiological function of apoptosis inhibition to prolong cell life. Bcl-2 can also protect cells because over expression of Bcl-2 causes the accumulation of nuclear glutathione (GSH), leading to changes in the nuclear redox balance, thereby reducing the activity of Caspase.

A study examined whether Lycium barbarum glycopeptide 3 (LBGP3) affects T cell apoptosis in aged mice. They found that treatment with 200 mg/mL LBGP3 increased the apoptotic rate of T cells from aged mice and showed a similar DNA ladder pattern to that in young T cells. The reversal of apoptotic resistance involved down-regulation of the expression of Bcl-2 and FLIP, and the up-regulation of Fas ligand (FasL). These results demonstrated that LBGP3 reverses apoptotic resistance of aged T cells by modulating the expression of apoptosis-related molecules [35]. Another study observed the protective effects of LBPs on focal cerebral ischemic reperfusion injury in mice. They found that neurological deficit scores were significantly reduced in the group receiving pretreatment of LBPs. Animal groups receiving LBPs (at 10, 20 and 40 mg/kg) showed dose-dependent reductions in neuronal damage through attenuation of neuronal apoptosis. Caspase-3 protein activity and Bcl-2-associated X (BAX) protein expression were clearly decreased, and Bcl-2 protein expression was markedly increased in animals receiving pretreatment of LBPs. Overall, this study provided evidence that LBPs offer protection against focal cerebral ischemic reperfusion injury in mice [36]. P53 is also an inhibitor of apoptosis. Studies have shown that the p53-mediated pathway likely mediates the effects of LBPs on cell apoptosis and aging. The expression of genes related to aging, such as p53, p21, and Bax showed decline after treatment with LBPs [37].

Previous clinical and epidemiological studies showed that homocysteine (Hcy) damages neurons by inducing apoptosis, DNA fragmentation, and tau hyperphosphorylation. A study showed that LBP treatment significantly attenuated Hcy-induced neuronal cell death and apoptosis in primary cortical neurons, as demonstrated by LDH and caspase-3. Treatment with LBPs suppressed elevation of both p-ERK and p-JNK, suggesting that LBPs exerted neuroprotective effects on cortical neurons exposed to Hcy [38].

LBP has been shown to reduce H_2_O_2_-induced cell apoptosis, the generation of ROS, the loss of Deltapsim, and the levels of MDA. LBP also inhibited H2O2-induced downregulation of Bcl-2 and up-regulation of Bax proteins and increased the levels of SOD and GSH enzyme activity. Moreover, LBP significantly attenuated H2O2-induced cellular senescence. Furthermore, LBPs protect human lens epithelial cells from H2O2-induced apoptosis by modulating the generation of ROS, loss of Deltapsim, Bcl-2 family of proteins, and antioxidant enzyme activity, and via attenuating cellular senescence [16].

### 1.4 LBPs reduce DNA damage

The information encoded in the nucleotide sequence of genomic DNA is essential for biological function. The genome of all living organisms is constantly threatened by a variety of agents which cause DNA damage. DNA damage is a vital challenge to cell homeostasis. Cells employ numerous mechanisms to repair damaged DNA and maintain genomic integrity. DNA lesions may occur by altering DNA bases (i.e. O6-methylguanine and thymine glycols), creating breaks on DNA backbone, and forming cross-links between DNA strands and proteins. Failure to repair these lesions can lead to genomic instability and detrimental consequences. Breaks on both strands of DNA (double-stranded break, DSB) represent one of the most lethal types of genomic lesion, which has been associated with pathogenesis of a variety of human diseases and aging [39, 40].

A study used human embryonic lung diploid fibroblasts 2BS national standard strain as an aging model to observe the effect of Wolfberry (WB) fruit and Epimedium (EM) on DNA synthesis. Both WB and EM accelerated the DNA synthesis rate of the aging 2BS fusion cells and prolonged their life span, likely through antagonizing the growth inhibitory factors [41]. Furthermore, LBPs were effective for reducing cellular DNA damage in peripheral lymphocytes of non-insulin-dependent diabetes mellitus (NIDDM) rats. LBPs decreased DNA damage through suppression of oxidative stress in this model [42].

## 2. Betaine

### 2.1 The Anti-aging effects of Betaine

Betaine is a natural amino-acid and one of the major functional components in Lycium barbarum. The Chinese Pharmacopoeia stipulates that Lycium barbarum contains not less than 0.3 % of Betaine. Betaine has been proven to have anti-aging effects [43]. Betaine is an essential nutrient for a human. Several studies have described its pharmacological action and noted the promise for disease prevention. It also has significant anti-aging effects [44].

#### 2.1.1 Betaine scavenges free radicals

Ultraviolet radiation B (UVB) is a common kind of free radicals that can cause skin aging. Betaine has been proved to reduce photodamage caused by UVB irradiation through the regulation of matrix metalloproteinase-9 activity in hairless mice [45]. When very-low-density lipoprotein (VLDL) oxalate into O-VLDL, it becomes another kind of free radicals, which damage the cardiovascular system and the liver. Dietary Betaine increases apolipoprotein B (ApoB) mRNA, VLDL ApoB, and triacylglycerol production and decreases hepatic triacylglycerol. Betaine likely mobilizes hepatic triacylglycerol by increasing ApoB available for VLDL assembly and secretion [46].

#### 2.1.2 Betaine reduces oxidative stress reaction

Betaine has anti-oxidant activity. For instance, it can inhibit the lipid peroxidation of RBC membrane, which is induced by H2O2 [47]. Betaine can mitigate carbon tetrachloride (CCl4)-induced hepatic injury by increasing antioxidative activity [48]. Betaine administration significantly suppressed the increase of alanine aminotransferase (ALT) and aspartate aminotransferase (AST) in the sera of CCl4 injured rats, restored the decreased levels of antioxidant enzymes such as total antioxidant capacity (TAC), SOD, CAT and GSH-Px, and suppressed the expression of inflammatory mediators including inducible NO synthase (iNOS) and cyclooxygenase (COX) -1 and -2.

Betaine has also been found efficacious for treating oxidative stress in some tissues of aged rats. Betaine could increase hepatic GSH and vitamin E levels, which are useful for decreasing oxidative stress in liver, heart and brain tissues [49]. Nuclear factor κB (NF-κB) is a nuclear transcription factor that regulates the expression of a vast number of genes that are critical for the regulation of apoptosis, viral replication, tumor genesis, inflammation, and various autoimmune diseases. It also plays an important role in antioxidant mechanisms.

In recent years, several studies on betaine-mediated antioxidant activities have focused on changes in the NF-κB pathway. Betaine has been reported to suppress the lysophosphatidylcholine (LPC) related AM expression associated with NF-κB activation via the up-regulation of an inhibitor of nuclear factor kappa-B kinase (IKK)/ mitogen-activated protein kinases (MAPKs). LPC is a mediator of endothelial dysfunction in the expression of adhesion molecules (AMs) during aging. Thus, it appears that betaine can prevent vascular disorders [50]. Betaine suppressed the age-related NF-κB activities associated with unregulated NF-κB -in NF-κB decibel (NIK)/IKK and MAPKs that were induced by oxidative stress. Thus, it might be useful as a preventive agent against the activation of NF-κB seen during inflammation and aging [51]. Betaine exerts its efficacy by maintaining thiol status in the regulation of COX-2 and TNF-alpha via NF-κB activation during aging [52]. Another investigation of NF-κB has shown that betaine was produced in the Ergothioneine (Egt) oxidation pathway. The cytoprotective effect of Egt was paralleled by reduced ROS production, cell senescence, and, interestingly, the formation of hercynine (EH), The Egt beneficial effect was exerted through the upregulation of sirtuin 1 (SIRT1) and sirtuin 6 (SIRT6) expression and the downregulation of p66Shc and NF-κB [53].

### 2.2 Betaine positively regulates mitochondrial respiration

Mitochondria are cellular respiration and energy metabolism center. Damage to mitochondria can lead to cell senescence and apoptosis. Mitochondrial electron transport chain (ETC) complexes generate the mitochondrial membrane potential, which is essential to produce cellular energy, adenosine triphosphate (ATP). Reduced mitochondrial respiration and energy status have been found in many human pathological conditions including aging, cancer, and neurodegenerative disease. Betaine has been proven to improve mitochondrial function contributing to a reversal of the Warburg effect; it leads to an upregulation of mitochondrial respiration and cytochrome oxidase activity in H2. 35 cells [54].

## 3. Beta-carotene (β-carotene)

Carotenoids are the second major group of metabolites of Lycium barbarum [55]. They contain zeaxanthin (83%), β-cryptoxanthin (7%), β-carotene (0.9%), and mutatoxanthin (1.4%), as well as some minor carotenoids which have not been definitively identified [56].

Carotenoids contain some conjugated double bonds, which can capture, quench free radicals and singlet oxygen: accordingly, it can reduce the damage of free radicals to cells [57]. Carotenoids and beta-carotene, in particularly, are important natural antioxidants. Singlet oxygen, the lowest excited state of molecular oxygen, is an intermediate often involved in natural oxidation reactions. The fact that beta-carotene efficiently quenches singlet oxygen in solution-phase systems is invariably invoked when explaining the biological antioxidative properties of beta-carotene [58]. One molecule beta- carotene can inhibit the activity of 1000 molecules of oxygen [59]. Beta- carotene can improve humoral immunity, cellular immunity, and non-specific immune responses. Beta- carotene can effectively increase the number of T cells and T lymph nodes and enhance the activity of NK [60]. Beta- carotene has a potential function of inducing apoptosis of cancer cells [61].

## 4. Zeaxanthin

Zeaxanthin (ZA) is xanthophyll carotenoid which is widely distributed in tissues, and is the principal carotenoid in the eye lens and macular region of the retina [62]. Zeaxanthin has 11 conjugated double bonds and ends with hydroxyl groups. Based on its molecular structure, Zeaxanthin has the high antioxidant capacity by quenching singlet oxygen and scavenging free radical. A significant amount of literature show that zeaxanthin mitigates visual problems and suppresses oxidative stress in the retinal tissues [63]. Zeaxanthin may retard the aging of the lens [64].

Zeaxanthin, an important compound found in Lycium barbarum, ZA inhibited the serum levels of inflammatory factors including interleukin-2 (IL-2), IL-6, tumor necrosis factor-α, and nuclear factor kappa B. Moreover, the serum imbalances in superoxide dismutase, glutathione peroxidase, methane dicarboxylic aldehyde, and catalase were normalized by ZA, suggesting its antioxidant properties [65].

Dietary Wolfberry on retinal protection in diabetic mice is, at least partially, due to zeaxanthin along with or in addition to lutein. The zeaxanthin preventive effect was abolished by small interfering RNA knockdown of AMPKα. AMPK activation appeared to play a vital role in upregulated expression of thioredoxin and Mn-SOD, and mitigation of cellular oxidative stress and/or ER stress by wolfberry and zeaxanthin and/or lutein [66].

Epidemiologic studies indicating an inverse relationship between xanthophyll intake or status and both cataracts and age-related macular degeneration suggest these compounds can play a protective role in the eye. Some observational studies have also shown the xanthophyll may help reduce the risk of certain types of cancer, particularly those of the breast and lung. Emerging studies suggest as well, a potential contribution of zeaxanthin to the prevention of heart disease and stroke [62].

## 5. 2-O-β-D-Glucopyranosyl-L-ascorbic acid

2-O-β-D-Glucopyranosyl-L-ascorbic acid (AA-2βG), another major component discovered and isolated from the fruits of Lycium barbarum, was characterized as a stable vitamin C analog with biological activities comparable to vitamin C. The content of AA-2βG in the Lycium barbarum fruit is up to 0.5% of the dry fruit, suggesting that the fruit is a natural plant resource for AA-2βG and could serve as a stable vitamin C substitute. Recently, 2-O-substituted AA derivatives per se have been found to have radical-scavenging activity. The AA derivatives per se act as biologically effective antioxidants under reduced oxidative stress [4, 67].

Several studies have evaluated the antioxidant activities of AA-2βG and AA using in vitro and in vivo model systems. In vitro, radical scavenging assays demonstrated that AA-βG was capable of scavenging 1, 1-diphenyl-2-picryl-hydrazyl and hydroxyl peroxide and inhibiting H_2_O_2_ -induced hemolysis better than AA. AA-2βG and AA had similar OH scavenging capabilities, but AA-2βG was incapable of scavenging O_2_- radicals, and its capacity to scavenge nitrite (NO_2_^-^) was lower than that of AA. The overall in vitro reduction capability of AA-2βG was also significantly lower than that of AA. Moreover, in vivo studies demonstrated that AA-2βG capable of protecting the liver against carbon tetrachloride-induced acute liver injury in mice. These results suggest that AA-2βG is an important antioxidant component of Goji berry fruit, which may share similar but distinct antioxidant mechanistic properties with AA [68].

Another report investigated the cytotoxic and antiproliferative effect of AA-2βG against cancer cells in vitro and identified the proteins with significantly differential expression in the cervical cancer cells (Hela) cultured in the presence of AA-2βG proteomic analysis. The results demonstrated that the cytotoxic and anti-proliferative activity of AA-2βG on cancer cell lines were in a cell type, time, and dose-dependent manner. Similar to vitamin C, the AA-2βG selectively induced cell death repressed the proliferation of Hela cells by the mechanism of cell apoptosis and cell cycle arrest induced by AA-2βG through a mechanism of stabilizing the p53 protein. These data indicate that a mechanism of the AA-2βG and vitamin C mediated antitumor activity by down-regulating the expression of proteins involved in cell apoptosis and proliferation and consequently inducing Hela cell apoptosis and cell cycle arrest, suggesting that AA-2βG and Vc may share a similar mechanism of inducing Hela cell apoptosis. These results also indicate that the Lycium barbarum fruit may be a potential dietary supplement and anticancer agent aimed at the prevention and treatment of cervical cancer [69].

## 6. Flavonoids

Flavonoids of Lycium Barbarum (L. TFL) are found in extracts of its leaves. TFL has the function of scavenging oxygen free radicals. It is an important antioxidant found in Lycium Barbarum. The mechanisms by which TFL mediates antioxidant activity may include scavenging of O_2_ and OH to block the chain reaction of lipid peroxidation, inhibition of the lipid peroxidation oxidation and detering the cell toxicity of MDA [70]. The structure that contributes most to the antioxidant capacity of flavonoids is B ring on the 3 ', 4' -O- two hydroxyl groups, C ring with 4- ketone group bound 2, 3- double bond and 3- hydroxyl group [71].

The protective effects of TFL on lipid peroxidation in mitochondria and red blood cells (RBC) induced by oxygen radicals produced by Fe^2+^ cysteine system were investigated. TFL significantly inhibited the mitochondrial lipid peroxidation (measured as MDA) in a dose-response relation between the concentrations of 0.025 and 2.0 mg/ml, and the fluidity of mitochondrial membrane was also protected effectively. Observation through a scanning electron microscope showed that the shape of RBC in the Fe2+ system was damaged significantly. However, the shape of RBC remained intact with the addition of TFL [70]. It is of interest to note that L. chinense leaves extract display a higher antioxidant activity than Lycium barbarum. Both Lycium barbarum and L. chinense leaves are valuable sources of flavonoids with important antioxidant and antimicrobial activities [72].

## 7. Cerebroside

Cerebroside belongs to the glycosyl sphingolipids. The structure of these compound contains a sugar group, two long chain alkyl groups and a group of amide bonds. Two cerebrosides isolated from Lycium barbarum fruits have been characterized as 1-O-beta-D-glucopyranosyl- (2S, 3R, 4E, 8Z) -2-N-palmitoyloctadecasphinga-4, 8-dienine+++ (LCC) and 1-O-beta-D-glucopyranosyl-(2S, 3R, 4E, 8Z) -2-N- (2'-hydroxy palmitoyl) octadecasph inga-4, 8-dienine. Pharmacological experiments showed that the two compounds could reduce the levels of glutamic pyruvic transaminase (GPT) and sorbitol dehydrogenase (SDH) released by injured cells [73]. Another study was conducted to determine the mechanism (s) by which LCC might exert its hepatoprotective activity. They postulate that LCC may preserve the hepatic mitochondrial level of GSHby scavenging ROS produced during CCl4-induced toxicity and thereby reduce lipid peroxidation and cellular damage [74].

## 8. Thiamine

Thiamine as the pentose phosphate pathway enzyme, transketolase coenzyme can activate transketolase enzyme to metabolize excess glucose through the pentose phosphate pathway, and reduce cytotoxic products and against apoptosis. Crude polysaccharide extracts of Lycium barbarum exhibited stronger antioxidant activity than purified polysaccharide extracts of Lycium barbarum because crude extracts were identified to be rich in antioxidants include thiamine [5].

Thiamine in phosphate forms can participate in many redox reactions in vivo, improve the energy metabolism of cells, scavenge oxygen free radicals, protect vascular endothelium against microvascular disease, avoid radiation-induced gene damage and play a role of anti-tumor and anti-mutation [75-78].

## 9. Riboflavin

Riboflavin has an antioxidant action independently or as a component of the glutathione redox cycle. Studies that examined the antioxidant properties of riboflavin and its effect on oxidative stress reduction are reviewed. Riboflavin can protect the body against oxidative stress, especially lipid peroxidation and reperfusion oxidative injury [79]. Quantitative real-time polymerase chain reaction analysis showed that riboflavin biosynthetic genes were constitutively expressed in all organs examined of Lycium Barbarum with the highest expression levels found in the leaves or red fruits [80].

Riboflavin as methylenetetrahydrofolate reductase (methylenetetrahydrofolate reductase, MTHFR) coenzyme-a flavin adenine dinucleotide (FAD) precursor, plays an important role in the folic acid metabolism and many redox reactions in vivo. This vitamin can reduce the genetic damage and cell damage, protect the cell's genetic stability and prevent the cell from malignant transformation [81].

Riboflavin can work against ischemia-reperfusion injury. By enhancing the capacity of scavenging free radical, such as increasing the activity of CAT, SOD, GSH-Px, and SDH, Riboflavin can reduce the production of free radical, then against the myocardial ischemia and reperfusion injury in vitro [82].

## 10. Beta-sitosterol

Beta-sitosterol (β-sitosterol) is one of the phytosterols. It belongs to the tetracycline compounds. It is widely found in various plant oils, nuts and other plant seeds in nature. In 1963, Beta-sitosterol was isolated from the leaves of Lycium barbarum [83] and from its roots [84]. It has been proved that β-sitosterol has some anti-oxidative effects in addition to the effect of lowering serum cholesterol. Therefore, it is of great practical significance to further study the antioxidant activity of β-sitosterol.

Studies have shown that β-sitosterol can prolong life, may activate the fat body by activating AMP protein kinase (AMPK), and has a strong free radical scavenging activity, can reduce paraquat-induced oxidative damage and mortality [85].

β-sitosterol can also be used as a plant hormone, can increase the weight of reproductive organs. Increasing the weight of the reproductive organs can trigger an anti-aging effect [86]. It has been shown that the hypothalamus plays an important role in the development of aging and that this effect of the hypothalamus is mediated by a decrease of the NF-κB -oriented gonadotropin-releasing hormone (GnRH). Further studies have shown that β-sitosterol can be incorporated into membranes to prevent TNF-α-induced activation of nuclear factor kappaB and decreased gonadotropin releasing hormone [87].

At the same time, it has been suggested that the number of peroxisomes in hepatocytes of young mice and aged mice of the β-sitosterol diet is significantly greater than that of peroxisomes in liver cells of those mice fed with the control diet quantity. β-sitosterol can also reduce cell death, with its role of anti-aging activity [88]. Also, lipid peroxidation is a process in which cell membranes are damaged by oxidative deterioration of poly-unsaturated lipids, which can lead to cell death and disease in living organisms. Substances such as vitamin E protect the cell membrane from oxidative damage due to their chemical structure. The structure of β-sitosterol and vitamin E is similar. In vitro studies have confirmed that β-sitosterol has a free radical scavenging ability [89].

## 11. Phenolics

Lycium barbarum have a high content of phenolics, including caffeic acid, p-coumaric acid, rutin, scopoletin, N-trans-feruloyl tyramine, and N-cis-feruloyl tyramine, an unreported N-feruloyl tyramine dimer was characterized as the most abundant polyphenol isolated from the berries [90]. The polyphenolic constituents may be responsible for the inhibition of lipid peroxidation and enhance the antioxidant activities of ethanol extract of Lycium barbarum [91].

Due to the presence of diorthohydroxyl aromatic moiety, caffeic acid is not only one of the most potent antioxidant phenyl propanoids but also display numerous other pharmacological effects ranging from anti-inflammatory to anticancer effects. Recent studies also demonstrated that caffeic acid both in its free form or conjugated with other groups such as quinic acid and sugars display profound implications in the brain including protection from toxicity induced by a variety of agents and/or experimental models of Alzheimer's disease. Besides the usual global antioxidant effects, caffeic acid also has specific anti-inflammatory mechanisms in the brain along with the various processes of β-amyloid formation, aggregation, and neurotoxicity targets [92].

p-Coumaric acid (4-hydroxycinnamic acid) is a phenolic acid which has numerous biological activities, including antioxidant, anti-cancer, antimicrobial, antivirus, anti-inflammatory, antiplatelet aggregation, anxiolytic, antipyretic, analgesic, and anti-arthritis activities, and their mitigatory effects against diabetes, obesity, hyperlipemia, and gout are compared [93].

Rutin, a polyphenolic bioflavonoid has shown a wide range of pharmacological applications due to its significant antioxidant properties. Conventionally, it is used as the antimicrobial, antifungal, and antiallergic agent. However, current research has shown its multispectrum pharmacological benefits for the treatment of various chronic diseases such as cancer, diabetes, hypertension, and hypercholesterolemia. Its use is advantageous over other flavonoids as it is a non-toxic and non-oxidizable molecule [94].

Scopoletin has antioxidant properties, anti-inflammatory activity, and spasmolytic action. It exerts apoptotic and antiproliferative effects on prostate cancer cell line [95]. Scopoletin is the active component of the fruit of L. barbarum for inhibiting PC3 cell proliferation [96]. Scopoletin has also proven to possess antioxidant activity [97]. The structure of scopoletin shows that the catechol group significantly contributes to the antioxidant activities of scopoletin [98]. Scopoletin could exert a positive effect on anti-aging related to autophagy through modulation of p53 in human lung fibroblasts. Furthermore, scopoletin enhances the level of transcription factors such as Nrf-2 and p-FoxO1 related to anti-aging. Also, scopoletin modulates the reprogramming proteins [99].

Seven major phenolics were isolated and identified of Lycium barbarum using NMR experiments. Six of such compounds turned out to be already known molecules. The remaining, and most abundant, antioxidant compound constituted an unreported molecule. It was identified as a dimer of N-feruloyltyramine. Hence, more in-depth studies on the bioactivities of N-feruloyl tyramine dimers need to be conducted, since the occurrence of such molecules might amplify the nutritive value of Lycium barbarum [90].

## Conclusion

Aging, defined as the accumulation of diverse deleterious changes in cells and tissues, is commonly associated with a reduction in physiological functions and is closely related to apoptosis. Harman proposed the free radical theory of aging that in the long-term evolution of an organism, free radicals in the body are at an equilibrium state, have a high degree of chemical reactivity, and cause lipid peroxidation. It leads to denaturation of nucleic acids, proteins, and other macromolecular components, resulting in the alteration and destruction of cell structure, eventually leading to aging and diseases [100]. However, endogenous antioxidant defense mechanism cannot be completely efficient, so dietary antioxidants are more important to prevent oxidative damage in the body. Chinese herbs have been widely applied in the field of medicine as anti-aging drugs because of few side-effects [101, 102]. Lycium barbarum, as a medicinal and edible material, can effectively clear O_2_-, reduce oxidative stress reaction and enhance the activity of antioxidant enzymes. It is meaningful that Lycium barbarum intake produced a nearly 10% increase in serum antioxidant capacities in human subjects under normal conditions [17]. Lycium barbarum can activate T cells, B cells, macrophages, NK cells and other major immune cells that regulate the body's cellular immune function and humoral immune function. One study shows that LBP can delay the atrophy of thymus and spleen of aging mice, thereby preventing the decline of cellular immune and humoral immune functions, and has the effect of slowing aging to a certain extent [103]. Meanwhile, it has been shown that LBPs could inhibit cell apoptosis and reduce DNA damage. An aging study model investigated the lifespan of drosophila and found, that LBPs can significantly prolong its lifespan [104]. Other constituents of Lycium barbarum, such as phenolics, AA-2βG, carotenoids (zeaxanthin and β-carotene), betaine, cerebroside, β-sitosterol, flavonoids, riboflavin, and thiamin also have significant antioxidant effects.

Therefore, Lycium barbarum intake is a useful and an attractive proposition for counteracting age-related oxidative stress. Lycium barbarum may also be used as a source of antioxidants in the nutraceutical, pharmaceutical, or cosmetic products. Meanwhile, a better understanding of its safety, pharmacokinetics, mechanisms of action, disposition pathways, and therapeutic targets will help with the optimal use. In summary, the traditional Chinese herb Lycium barbarum is a promising anti-aging agent [105].
